# The severity of moral distress in nurses: a systematic review and meta-analysis

**DOI:** 10.1186/s13010-022-00126-0

**Published:** 2022-11-09

**Authors:** Nader Salari, Shamarina Shohaimi, Behnam Khaledi-Paveh, Mohsen Kazeminia, Mohammad-Rafi Bazrafshan, Masoud Mohammadi

**Affiliations:** 1 Department of Biostatistics, School of Health, Kermanshah University of Medical Sciences, Kermanshah, Iran; 2 Department of Biology, Faculty of Science, University Putra Malaysia, Serdang, Selangor Malaysia; 3 Sleep Disorders Research Center, Kermanshah University of Medical Sciences, Kermanshah, Iran; 4 Student research committee, Kermanshah University of Medical Sciences, Kermanshah, Iran; 5 Department of Nursing, School of Nursing, Larestan University of Medical Sciences, Larestan, Iran; 6 Cellular and Molecular Research Center, Gerash University of Medical Sciences, Gerash, Iran

**Keywords:** Moral distress, Nurses, Frequency, Severity, Meta-analysis

## Abstract

**Background:**

Moral distress is one of the most important problems that nurses face in their care of patients. Various studies have reported the frequency and severity of moral distress in nurses. However, to date, a comprehensive study that shows the results of these research across the world was not found, therefore due to the importance of this issue, its role in the health of nurses and patients, and the lack of general statistics about it worldwide, the present study was conducted to determine the frequency and severity of moral distress in nurses through a systematic review and meta-analysis.

**Methods:**

In this review study, searching national and international databases of SID, MagIran, IranMedex, IranDoc, Google Scholar, Embase, ScienceDirect, Scopus, CINHAL, PubMed, and Web of Science (WoS) between 2005 and February 2020 were extracted. The random-effects model was used for analysis, and the heterogeneity of studies with the I^2^ index was investigated. Data were analyzed using Comprehensive Meta-Analysis (Version 2).

**Results:**

The frequency of moral distress in 9 articles with a sample size of 1576 persons was 1.7 ± 0.5 from (0–4), in 13 articles with a sample size of 1870 persons, 3.07 ± 0.1 from (0–5), in 6 articles with a sample size of 1316 persons, 3.2 ± 0.29 from (0–6), in 18 articles with a sample size of 1959 persons, 4.6 ± 0.518 from (1–7) and in 35 articles with a sample size of 3718 persons, 81.1 ± 4.6 from (216–30), and the severity of moral distress in 4 articles with a sample size of 1116 persons, 1.7 ± 0.37 from (0–4), in 5 articles with a sample size of 1282 persons, 2.6 ± 0.28 from (0–5), in 5 articles with a sample size of 944 persons, 3.9 ± 0.63 from (0–6) and in 8 articles with a sample size of 901 persons was 82.3 ± 5.4 (0–216).

**Conclusion:**

The results of this study showed that the frequency and severity of moral distress in nurses are high and are a serious problem in nurses. Therefore, policymakers in this field should consider its role in the health of nurses and patients.

## Background

Moral distress is one of the most important problems that nurses face in their care of patients [1]. Nurses are at high risk of emotional conflict as a result of repeated exposure to large numbers of sick people and their mortality, and ethics play an important role in this profession [2]. Moral practice is a vital aspect of nursing care, and the development of moral competence is essential for the present and future of nursing [3]. There is the possibility of moral distress in a person acting contrary to their moral beliefs [4].

Moral distress is one of the significant dimensions of moral conflict, which has devastating effects on health care organizations through its impact on organizational culture, quality of care, and success in care [5, 6]. Professor Jamton (1984) defined moral distress as painful feelings or an imbalance of mental peace. This occurs when nurses are unable to convert their ethical choices or norms into ethical practices [1]. Wilkinson considers moral distress a psychological imbalance and a state of negative emotions in which one’s moral decision does not lead to moral action [7].

Various studies have been conducted on the concept of moral distress, the implications, and strategies for overcoming moral distress, including qualitative and quantitative studies. Researchers such as Corley, Redman, and Wilkinson have studied the levels of moral distress and prevalence of this phenomenon in nurses and reported different levels of moral distress. Corley designed the scale of moral distress in 2000 using the concepts proposed by Hauss and Rizzo [8–10].

Ineffectiveness of care services for the patient and prolonging patient suffering, performances out of regulations of clinical specialists, inadequacies of colleagues, lack of knowledge of nurses in patient care, lack of administrative support including salaries, working hours, benefits, and unacceptable working conditions such as inadequate management, overwork, and lack of proper support from managers are powerful sources of moral distress [2, 11]. Symptoms of ethical distress in various investigations include failure to provide good and effective physical care, reduced patient care, avoiding eye contact with the patient, and problems such as lack of sleep, overeating, poor social communication, reduced cooperation, defensive action, decreasing confidence and decreasing job satisfaction in nurses [6, 12, 13].

Nurses face more moral stress due to the complexity of care and increasing expectations [12]. A study on nurses of cardiovascular, internal, and neuroscience departments experiences greater moral distress, and this experience is directly correlated with burnout and inversely correlated with psychological hardiness [2]. A study in Iran reported that many nurses experience ethical distress due to cases such as lack of support from nurses, disrespect for patient rights, lack of professional and functional qualifications of physicians, and unnecessary tests for patients, and nurses of intensive care units experience moral distress more than other departments [14].

Although researchers with different tools have studied moral distress at different time intervals and in different departments, they cannot compare the results of the studies. However, the results can emphasize the necessity and the importance of this ethical dilemma. Among the tools used in investigating moral distress are the Jameton (1984), Corley (2007, 2001), and Hammer (2007) questionnaires; each one is different in terms of number and type of options [15].

Various studies have reported the frequency and severity of moral distress in nurses. But a comprehensive study that shows the results of these researches globally was not found. Therefore due to the importance of this issue, its role in the health of nurses and patients, and the lack of general statistics about it worldwide, the present study was conducted to determine the frequency and severity of moral distress in nurses through a systematic review and meta-analysis.

## Method

This systematic review and meta-analysis study investigated the frequency and severity of moral distress in nurses based on studies conducted between 2005 and February 2020. For this purpose, articles published in the databases of SID, MagIran, IranMedex, and IranDoc and Google Scholar and international databases Embase, ScienceDirect, Scopus, PubMed and Web of Science (WoS), and CINHAL with the keywords Moral Distress, Nurses, Intensity and Frequency were searched.

### Selection of studies

First, all articles that referred to the frequency and severity of moral distress in nurses were collected by researchers, and studies were accepted based on inclusion and exclusion criteria. The criteria for selecting the studies were the availability of the full text that examined the frequency and severity of moral distress in nurses. For access to more information, the sources of the articles reviewed were reviewed for access to other articles.

Exclusion criteria included unrelated cases with the issue, case reports, interventional studies, duplication of studies, unclear methodology, and inaccessibility of the full text of the study. In order to reduce bias, the articles were searched independently by two researchers, and in case of disagreement in the study, the article was reviewed by the referee. Sixty studies entered the third stage, i.e., qualitative evaluation.

### Qualitative evaluation of studies

The quality of the articles was evaluated based on the selected and related items of the STROBE 22- item checklist that could be assessed in this study (study design, literature review, place and time of study, outcome, inclusion criteria, sample size, and statistical analysis) that in previous studies, they have been referred. Articles referring to 6 to 7 criteria were considered high-quality articles. Articles that did not refer to 2 items or more from the seven items were considered medium and low methodological quality articles [16]. In the present study, 56 articles were included in the systematic review and meta-analysis as high-quality and medium-quality studies, and 4 articles had low quality and were eliminated.

### Extracting the data

All final articles entered into the meta-analysis process were extracted by a pre-prepared checklist. The checklist included the article title, first author’s name, year of publication, place of study, sample size, frequency and severity of moral distress in nurses, and method. A group of articles used the moral distress questionnaire, first developed by Jameton in 1984 and revised in 2001 and 2007 by Corley and 2007 by Humrick. This tool consisting of 21 items, measures the frequency and severity of moral distress on a four-point Likert scale in frequency dimensions ranging from never (equivalent to zero) to daily (equivalent to 4) and in severity dimensions from never (equivalent to zero) to very high (equivalent to 4) [17]. The next group of articles used the moral distress questionnaire, first developed in 1995 by Corley et al. This tool consisting of 21 items, measures the frequency and severity of moral distress based on a five-point Likert scale in frequency dimensions ranging from never (equivalent to zero) to daily (equivalent to 5) and in severity dimension from never (equivalent to zero) to very high (equivalent to 5) [18]. The third group of articles used the moral distress questionnaire of Corley et al. (2005). This tool consisting of 38 items, measures the frequency and severity of moral distress based on a three-point Likert scale in frequency dimension ranging from never (equivalent to zero) to daily (equivalent to 6) and in severity dimension from never (equivalent to zero) to very high (equivalent to 6) [19].

The fourth group used the Jamiton moral distress scale. The questionnaire consisted of 30 questions with answers on a 7-point Likert scale. The number 1 was the lowest stress, and the number 7 was the highest stress [14]. The fourth group of the Corley moral distress questionnaire has 36 questions and investigates the severity of moral distress based on nurses’ clinical situations. The items of this questionnaire are measured based on a Likert scale from 0 to 6. On this scale, zero indicates a lack of moral distress, and number 6 indicates severe moral distress. The total score of the questionnaire is between 0 and 216 [20].

### Statistical analysis

Since the frequency and severity of moral distress in nurses have binomial distribution, frequency and severity variance were calculated using the variance formula of the binomial distribution. A weighted average was used to combine the frequency and severity of moral distress in nurses. In order to evaluate the heterogeneity of the selected studies, the I^2^ index test was used (heterogeneity was divided into three classes less than 25% (low heterogeneity), 25–75% (moderate heterogeneity), and more than 75% (high heterogeneity)). Egger’s test at the significant level of 0.05 and its corresponding Funnel plot was used to investigate the publication bias and consider the high volume of samples entering the study. Sensitivity analysis was used to evaluate the effect of each of the studies on the final result. Data were analyzed using Comprehensive Meta-Analysis (Version 2) software.

## Results

**Fig. 1:**
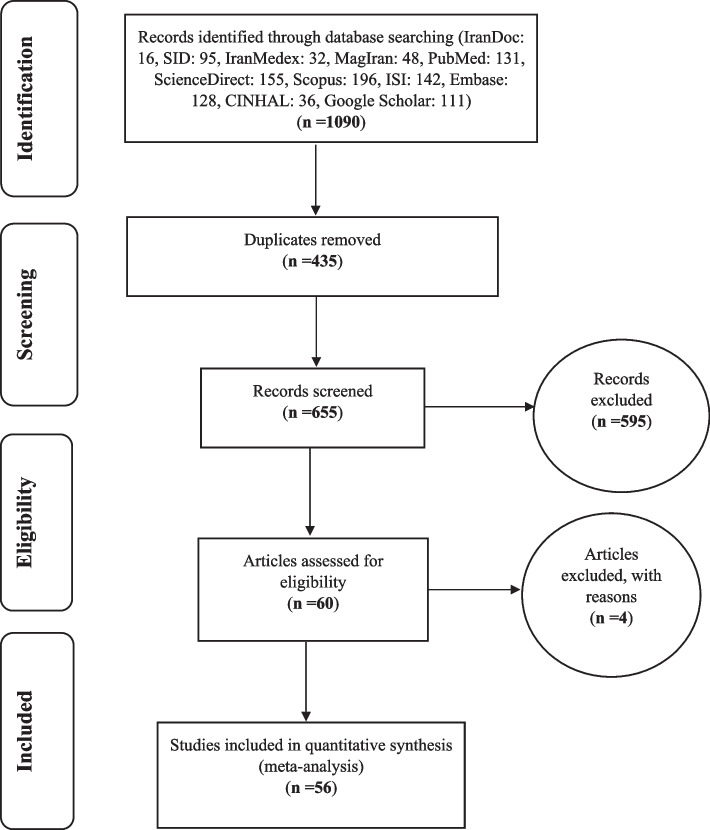
Flow diagram of study selection

All studies on the frequency and severity of moral distress in nurses were systematically investigated without time limitation and based on PRISMA guidelines. In the initial search, 943 articles were identified; finally, 56 articles were published between 2005 and February 2020 and entered the final analysis (Fig. 1).

Investigating the heterogeneity of the results of studies on the frequency of moral distress in the groups (0–4), (0–5), (0–6), (1–7), and (0–216) was reported (I^2^: 99.9), (I^2^: 99.7), (I^2^: 99.5), (I^2^: 99.9), and (I^2^: 99.8)) and in the severity of moral distress in groups (0–4), (0–5), (0–6), (1–7) and (0–216) reported (I^2^: 99.8), (I^2^: 99.3), (I^2^: 99.6), (I^2^: 95.1), and (I^2^: 98.7) and according to the heterogeneity of studies selected, a random-effects model was used to combine studies and to estimate frequency and severity. There were 56 articles with sample size, frequency, and severity of ethical distress, and the method in each study and selected articles’ characteristics have been presented in (Table 1).

**Table 1:** Characteristics of meta-analysis studies on the Moral Distress Nurses

Author, year, [Reference]	Country	Sample size	frequency	intensity	Score Scale	Service location	Quality
Mahdavi, 2017, [21]	Iran	125	1.4 ± 0.58	1.7 ± 0.92	0–4	Emergency	High
Robaee, 2018, [22]	Iran	120	2.19 ± 0.58	–	0–4	Health centers	High
O’Connell, 2014, [23]	USA	31	1.71	–	0–4	Health centers	High
Fernandez-Parsons, 2013, [24]	USA	61	3.18	–	0–4	Health centers	High
Piers, 2011, [25]	Belgium	222	1.05 ± 0.65	2.26 ± 1.17	0–4	Elderly nurse	Medium
Pauly, 2009, [26]	Canada	38	1.31 ± 0.72	–	0–4	Health centers	High
Ameri, 2012, [27]	Iran	148	2.13 ± 0.44	2.08 ± 0.36	0–4	oncology	High
Jolaei, 2013, [28]	Iran	210	1.77 ± 0.91	–	0–4	ICU, CCU, Emergency	High
Laurs, 2019, [29]	Lithuania	621	0.57	1.02	0–4	Health centers	Medium
Borhani-1, 2015, [30]	Iran	160	4.25 ± 0.25	–	0–5	ICU	Medium
Borhani-2, 2015, [30]	Iran	53	3.0 ± 0.88	–	0–5	CCU	High
Borhani-3, 2015, [30]	Iran	35	3.99 ± 0.50	–	0–5	NICU	High
Borhani-4, 2015, [30]	Iran	32	2.6 ± 0.72	–	0–5	Oncology	High
Borhani-5, 2015, [30]	Iran	20	2.8 ± 0.65	–	0–5	Dialysis	High
Sarkoohijabalbarezi, 2016, [31]	Iran	120	2.68 ± 0.75	–	0–5	Children’s sections	High
Saleh, 2018, [32]	Iran	172	0.62 ± 2.01	0.89 ± 3.11	0–5	NICU	Medium
Jeon-1, 2019, [33]	Korea	96	3.42	–	0–5	end-of-life care	High
Jeon-2, 2019, [33]	Korea	72	3.16	–	0–5	Burn	High
Mohammadi-1, 2014, [34]	Iran	260	3.9 ± 0.55	3.5 ± 0.8	0–5	Health centers	High
Mohammadi-2, 2014, [35]	Iran	330	3.7 ± 1.2	3.5 ± 0.9	0–5	Health centers	Medium
AbbasZadeh, 2013, [36]	Iran	220	2.11 ± 0.56	2.25 ± 0.6	0–5	Health centers	High
Mohammadi-Nafchi, 2013, [37]	Iran	300	3.24 ± 0.43	2.7 ± 0.57	0–5	Health centers	High
Dyo, 2016, [38]	USA	426	2.6 ± 0.11	2.5 ± 0.19	0–6	ICU, NICU	High
Hamaideh, 2014, [39]	Jordan	130	4.89 ± 2.04	–	0–6	mental health nurses	High
Ohnishi, 2010, [40]	Japan	264	4.89 ± 2.04	4.03 ± 1.79	0–6	mental health nurses	High
Pauly, 2009, [26]	Canada	38	–	5.63 ± 0.69	0–6	Health centers	High
Zuzelo, 2007, [41]	USA	97	2.3 ± 1.65	3.64 ± 2.19	0–6	Health centers	High
Rathert, 2015, [42]	USA	280	3.13 ± 1.32	–	0–6	Health centers	High
DeKeyser, 2011, [43]	Israel	119	1.8 ± 0.4	3.80 ± 1.0	0–6	ICU	Medium
Tavakol, 2014, [44]	Iran	60	4.5 ± 0.88	–	1–7	CCU	High
Abaszadeh, 2012, [45]	Iran	140	5.041 ± 0.879	–	1–7	Health centers	High
Burton, 2020, [46]	USA	57	5.25	–	1–7	NICU	High
Abbasi, 2018, [47]	Iran	13	5.12 + 2.70	–	1–7	ICU	High
Vaziri-1, 2015, [48]	Iran	111	5.06 ± 0.179	–	1–7	CCU	Medium
Vaziri-2, 2015, [48]	Iran	89	4.96 ± 0.091	–	1–7	ICU	High
Vaziri-3, 2015, [48]	Iran	107	3.95 ± 1.516	–	1–7	Internal	Medium
Vaziri-4, 2015, [48]	Iran	93	5.0 ± 0.097	–	1–7	Surgery	High
Vaziri-5, 2015, [48]	Iran	125	4.50 ± 0.280	–	1–7	NICU	High
Vaziri-6, 2015, [48]	Iran	141	5.0 ± 0.169	–	1–7	Operating room	High
Vaziri-7, 2015, [48]	Iran	88	5.26 ± 0.259	–	1–7	EMS	High
Vaziri-8, 2015, [48]	Iran	73	5.08 ± 0.416	–	1–7	ENT	High
Vaziri-9, 2015, [48]	Iran	95	4.37 ± 0.391	–	1–7	Ophthalmology	High
Dalmolin, 2014, [49]	Brazil	334	3.69	–	1–7	Health centers	High
Mason, 2014, [50]	USA	26	3.8 ± 0.8	–	1–7	ICU	Medium
Molazem, 2013, [51]	Iran	30	4.57 ± 1.03	–	1–7	CCU	Medium
Browning, 2013, [52]	USA	227	5.34 ± 1.32	–	1–7	ICU	High
Elpern, 2005, [53]	USA	100	3.94	5.15	1–7	ICU	Medium
Etebari, 2016, [54]	Iran	118	108.25 ± 24.18	–	0–216	ICU	High
Behbodi-1, 2018, [55]	Iran	60	37.48 ± 36.39	–	0–216	Children’s sections	High
Behbodi-2, 2018, [55]	Iran	51	58.02 ± 36.47	–	0–216	NICU	High
Shafipour, 2015, [56]	Iran	172	–	105.65 ± 52.39	0–216	Burn Sections	High
Mardani-1, 2016, [57]	Iran	114	136 ± 4.8	–	0–216	Health centers	High
Mardani-2, 2016, [57]	Iran	81	124 ± 4.2	–	0–216	Health centers	High
Meziane-1, 2018, [58]	Canada	19	107.97 ± 59.37	–	0–216	End-of-life care	High
Meziane-2, 2018, [58]	Canada	16	100.28 ± 54.67	–	0–216	End-of-life care	High
Asayesh, 2018, [59]	Iran	117	76.48 ± 13.57	74.45 ± 11.08	0–216	ICU	High
Hatamizadeh-1, 2018, [60]	Iran	223	40.87 ± 10.41	63.19 ± 15.95	0–216	Health centers	High
Hatamizadeh-2, 2018, [60]	Iran	53	40.32 ± 10.80	66.56 ± 12.54	0–216	Health centers	High
Ajoudani, 2019, [61]	Iran	278	91.02 ± 35.26	–	0–216	Health centers	High
Ghasemi, 2019, [62]	Iran	125	106.41 ± 61.64	–	0–216	Children’s sections	High
Asgari, 2019, [63]	Iran	142	87.02 ± 44.56	–	0–216	ICU	High
Sirilla, 2017, [64]	USA	134	94.97 (44.57 to 134.58)	–	0–216	ICU	High
Soleimani-1, 2016, [65]	Iran	12	95.83 ± 16.38	–	0–216	CCU	High
Soleimani-2, 2016, [65]	Iran	58	98.72 ± 14.69	–	0–216	ICU	High
Soleimani-3, 2016, [65]	Iran	20	91.85 ± 12.89	–	0–216	Emergency	High
Soleimani-4, 2016, [65]	Iran	48	92.52 ± 15.11	–	0–216	Medical	High
Soleimani-5, 2016, [65]	Iran	20	90.5 ± 17.25	–	0–216	Surgical	High
Soleimani-6, 2016, [65]	Iran	6	82.16 ± 15.21	–	0–216	Pediatric	High
Soleimani-7, 2016, [65]	Iran	3	84.66 ± 28.37	–	0–216	Dialysis	High
Soleimani-8, 2016, [65]	Iran	8	98.75 ± 14.61	–	0–216	Nursing office as superviso	High
Soleimani-9, 2016, [65]	Iran	18	99.0 ± 18.83	–	0–216	Operation room	High
Zavotsky, 2016, [66]	USA	198	80.19 ± 53.27	–	0–216	Emergency	High
Borhani, 2017, [67]	Iran	153	44.8 ± 16.6	–	0–216	ICU	Medium
Karagozoglu, 2017, [68]	Turkey	200	70.81 ± 48.23	–	0–216	ICU	High
Woods, 2014, [69]	New Zealand	412	51.91 ± 36	–	0–216	Health centers	High
De Villers -1, 2014, [70]	USA	66	85.71 ± 23.42	143.99 ± 57.45	0–216	CCN	High
De Villers -2, 2014, [70]	USA	28	79.86 ± 20.37	129.21 ± 54.48	0–216	nCCN	High
Papathanassoglou, 2012, [71]	England	225	73.67 ± 39.19	–	0–216	ICU	High
Abdolmaleki, 2018, [72]	Iran	187	47.49 ± 14.01	49.32 ± 16.80	0–216	Health centers	Medium
Trautmann, 2015, [73]	USA	246	74.4 ± 39.6	–	0–216	Emergency	High
Hamric, 2007, [74]	USA	29	44.61 ± 22.59	–	0–216	ICU	High
Saeedi, 2018, [75]	Iran	55	49.02 + 19.21	53.65 + 12.18	0–216	ICU	High

Publication bias in the distribution of distress frequency results in groups (0–4), (0–5), (0–6), (1–7) and (0–216) by funnel plot and Egger’s test at the significant level of 0.05 0 indicated no emission bias in the present study (*P* = 0.275), (*P* = 0.856), (*P* = 0.640), (*P* = 0.054), and (*P* = 0.310).

Publication bias in the distribution of severity of moral distress results in groups (0–4), (0–5), (0–6), (1–7), and (0–216) by funnel plot and Egger’s test at the significant level of 0.05 0 indicated no distribution bias in the present study (*P* = 0.147), (*P* = 0.946), (*P* = 0.053), (*P* = 0.159).

**Fig. 2:**
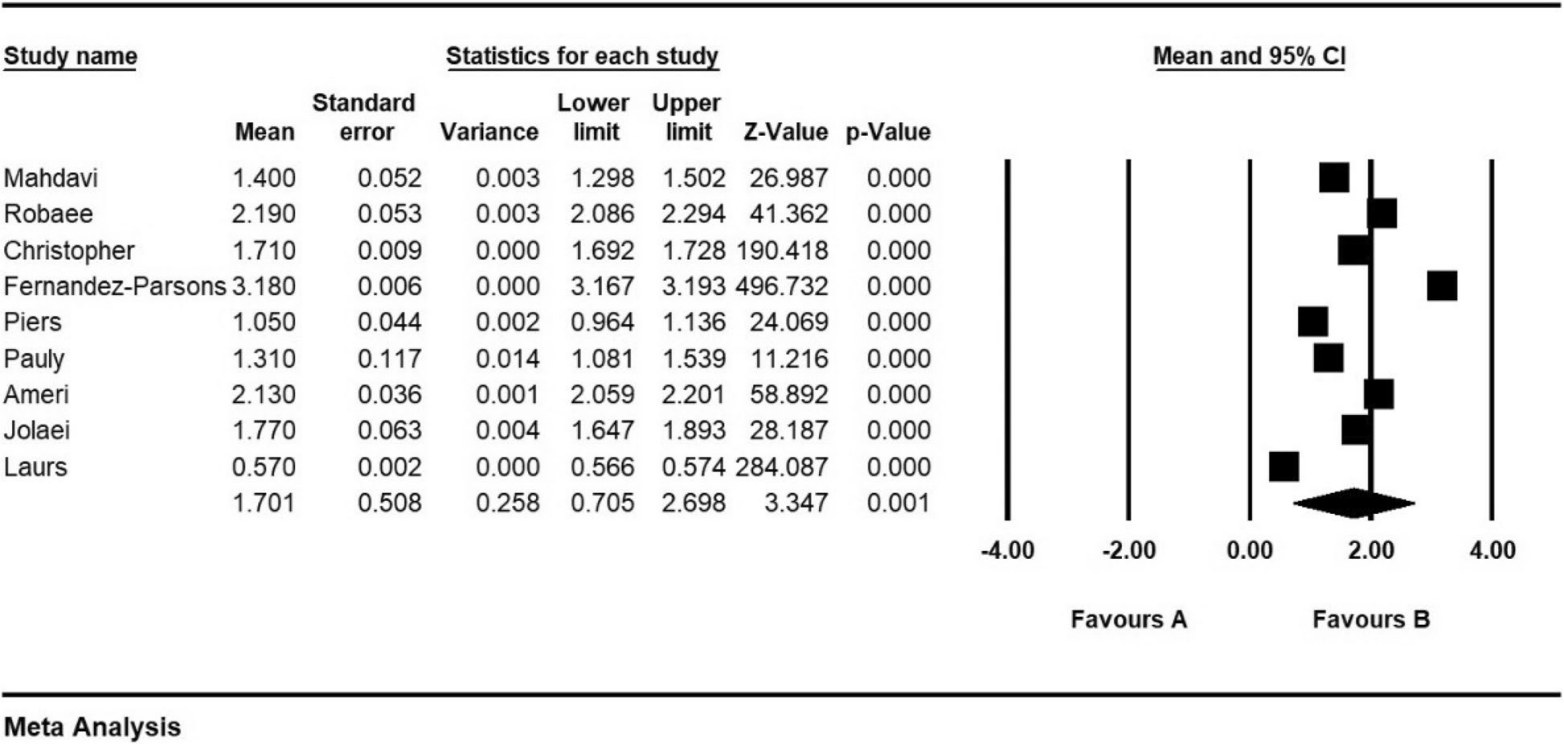
Frequency of moral distress in nurses and 95% confidence interval in the group (0–4). The middle point of each line segment shows the frequency of moral distress in each study, and the diamond shape shows the frequency of moral distress in nurses for the entire study

**Fig. 3:**
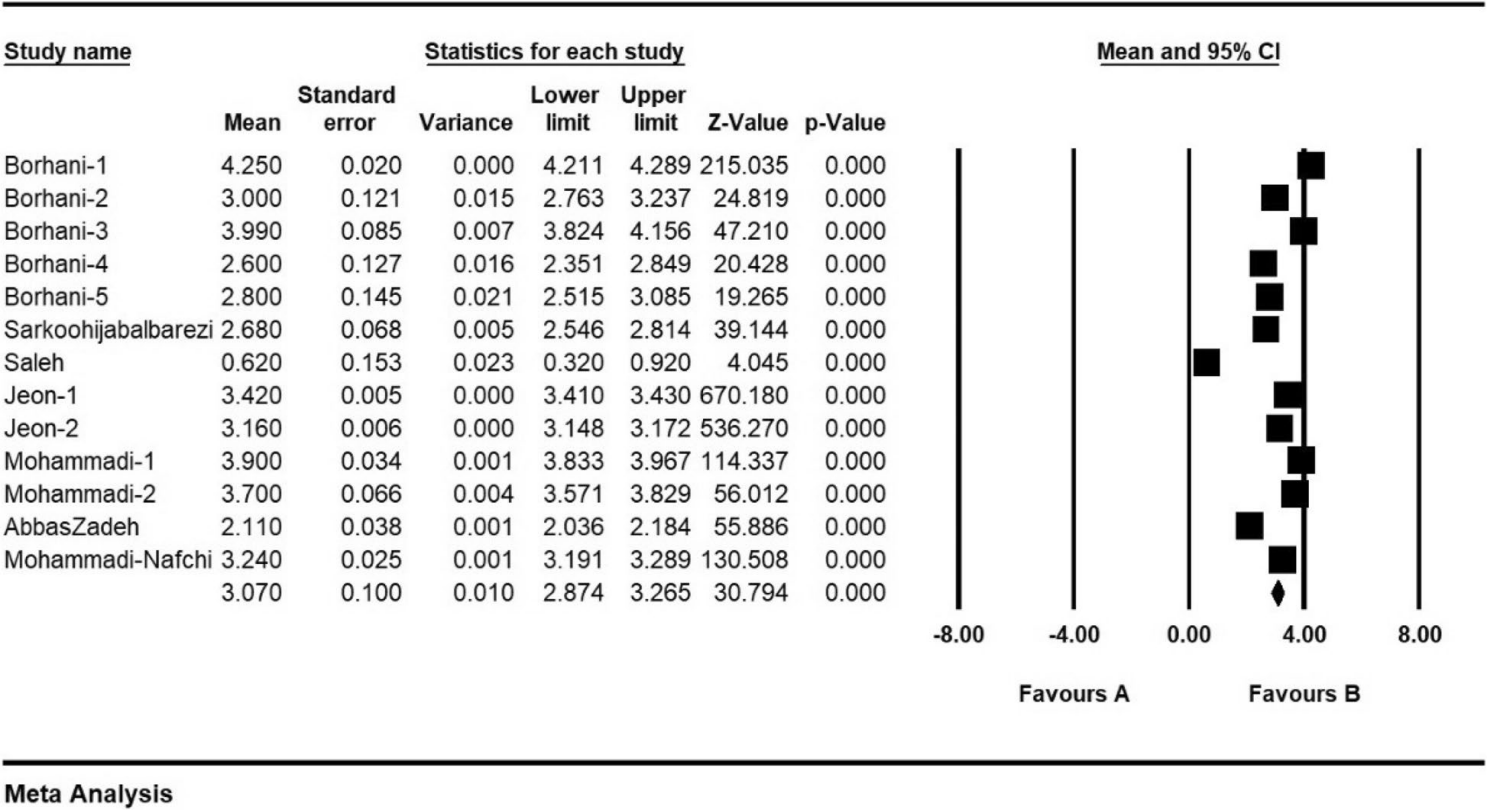
Frequency of moral distress in nurses and 95% confidence interval in the group (0–5). The middle point of each line segment shows the frequency of moral distress in each study, and the diamond shape shows the frequency of moral distress in nurses for the entire study

**Fig. 4:**
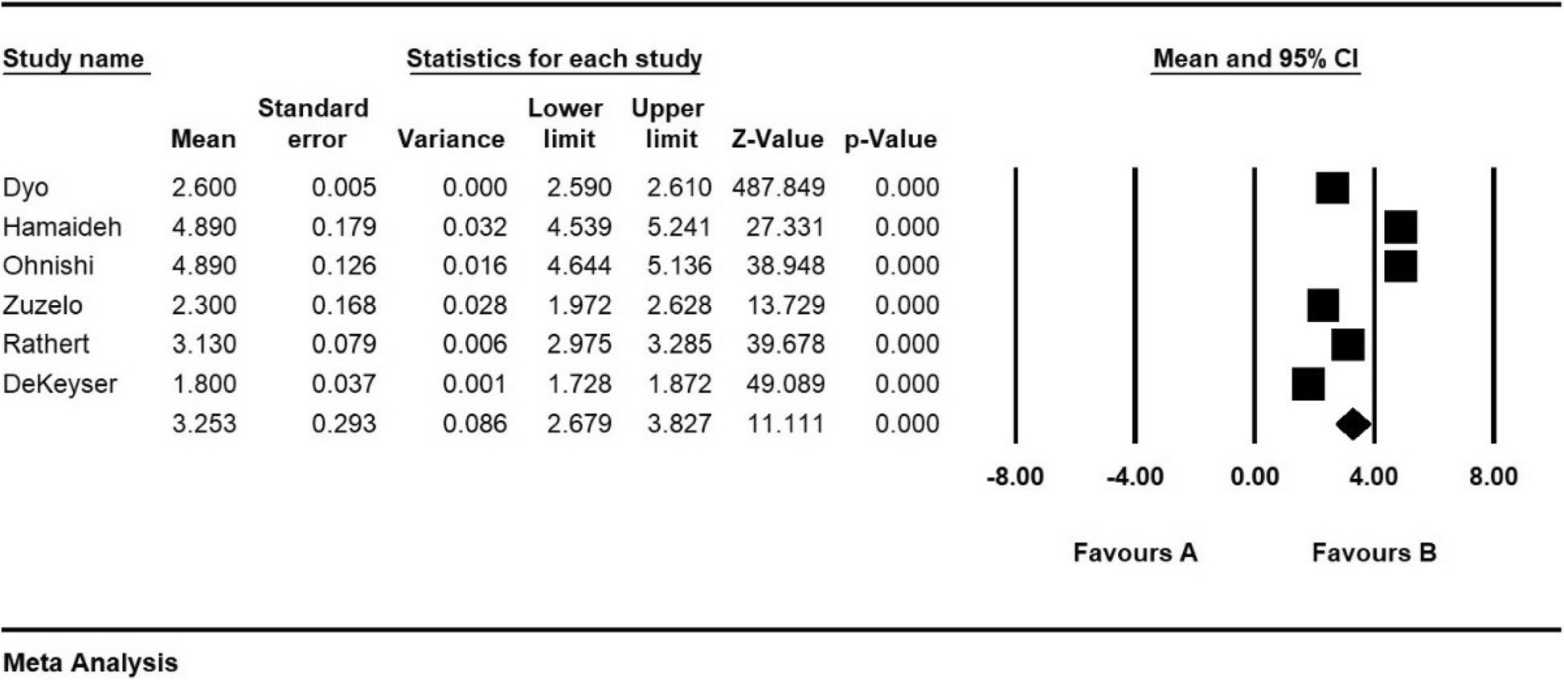
Frequency of moral distress in nurses and 95% confidence interval in the group (0–6). The middle point of each line segment shows the frequency of moral distress in each study, and the diamond shape shows the frequency of moral distress in nurses for the entire study

**Fig. 5:**
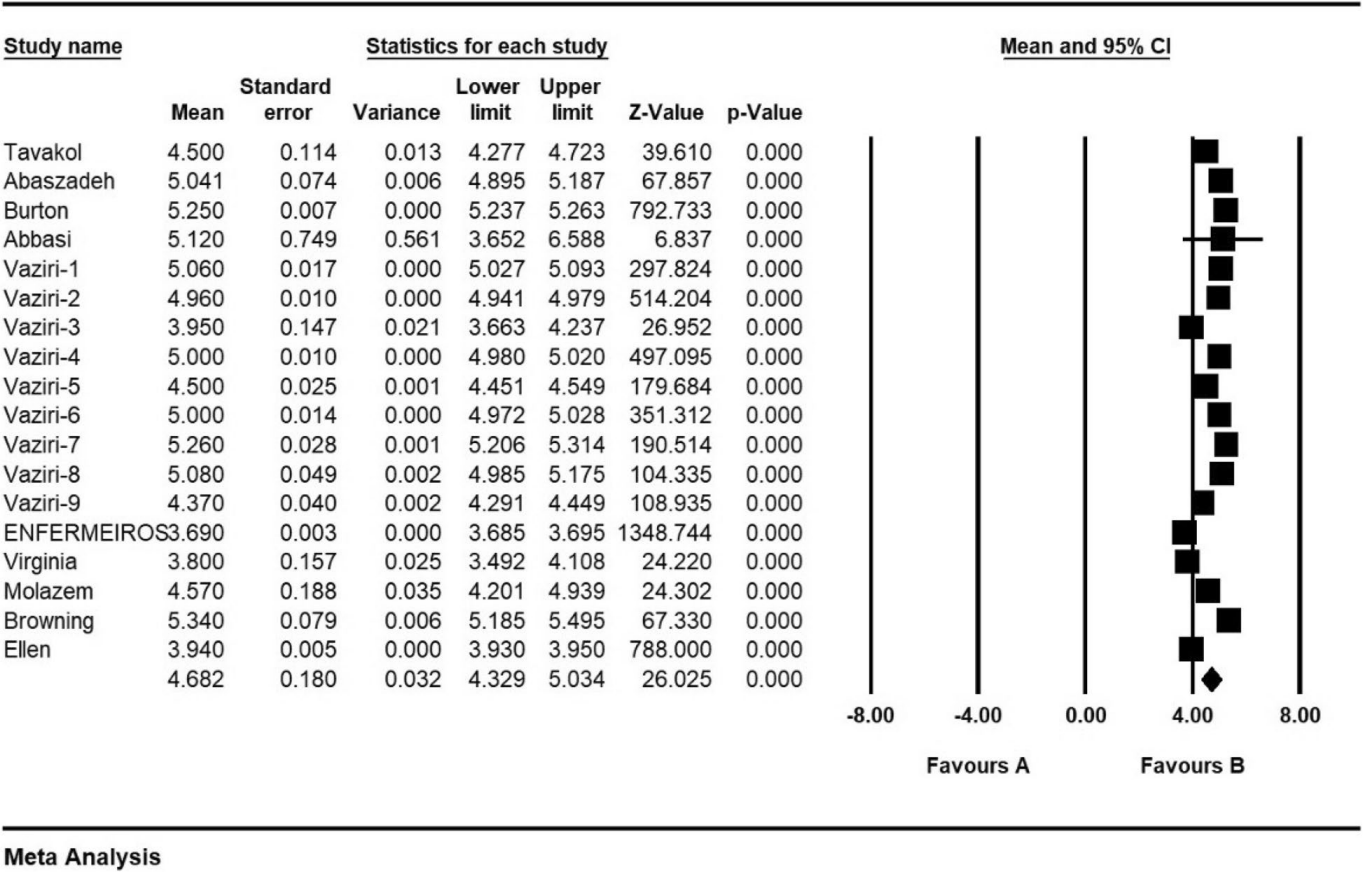
Frequency of moral distress in nurses and 95% confidence interval in the group (1–7). The middle point of each line segment shows the frequency of moral distress in each study, and the diamond shape shows the frequency of moral distress in nurses for the entire study

**Fig. 6:**
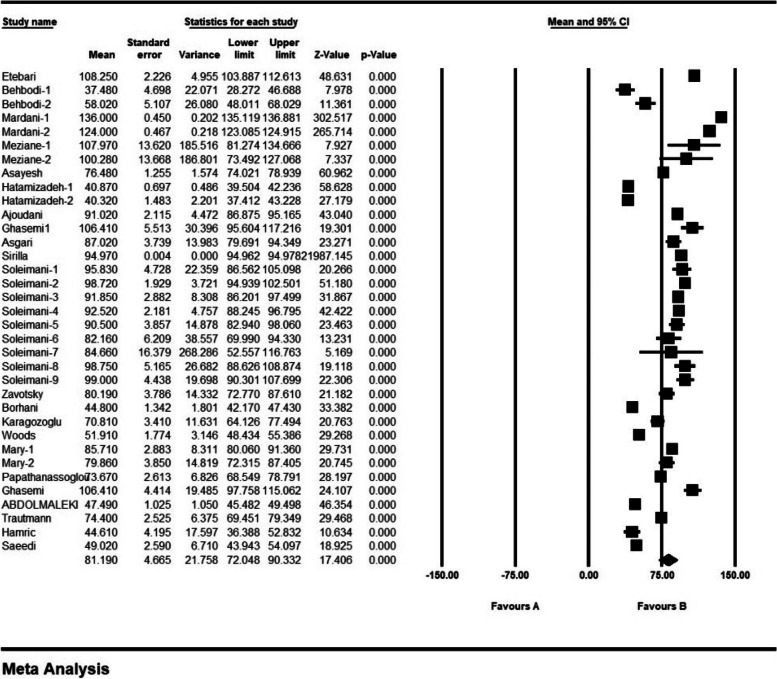
Frequency of moral distress in nurses and 95% confidence interval in the group (0–216). The middle point of each line segment shows the frequency of moral distress in each study, and the diamond shape shows the frequency of moral distress in nurses for the entire study

The frequency of ethical distress in 9 articles with a sample size of 1576 persons was 1.7 ± 0.5 from (0–4); the frequency of ethical distress in 13 articles with a sample size of 1870 persons was 3.07 ± 0.1 from (0–5); the frequency of ethical distress in 6 articles with a sample size 1316 persons was 3.2 ± 0.29 from (0–6); the frequency of ethical distress in 18 articles with sample size 1959 persons was 4.6 ± 0.518 from (1–7); and the frequency of ethical distress in 35 articles with a sample size of 3718 persons was 81.1 ± 4.6 from (0–216) (Figs. 2, 3, 4, 5 and 6).

**Fig. 7:**
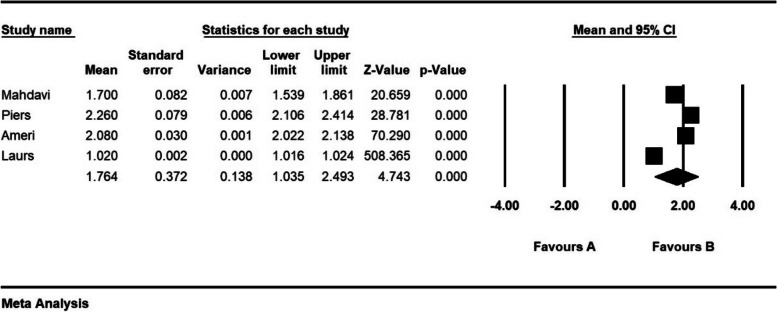
Severity of moral distress in nurses and 95% confidence interval in the group (0–4). The middle point of each line segment shows the severity of moral distress in each study, and the diamond shape shows the severity of moral distress in nurses for the entire study

**Fig. 8:**
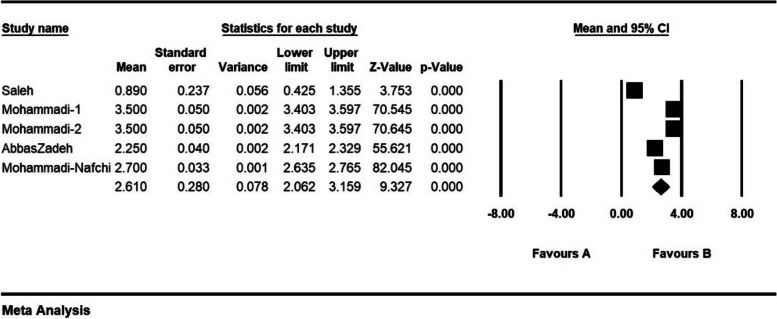
Severity of moral distress in nurses and 95% confidence interval in the group (0–5). The middle point of each line segment shows the severity of moral distress in each study, and the diamond shape shows the severity of moral distress in nurses for the entire study

**Fig. 9:**
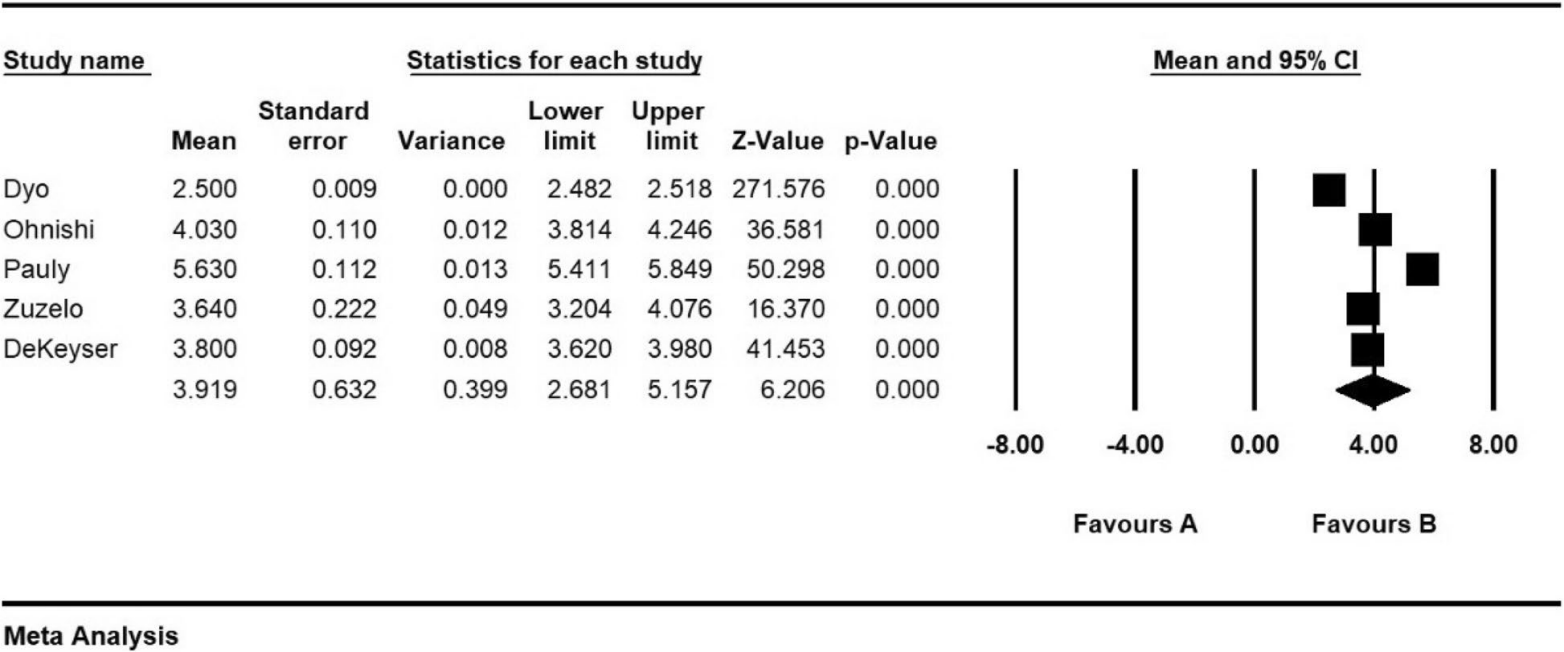
Severity of moral distress in nurses and 95% confidence interval in group (0–6). The middle point of each line segment shows the severity of moral distress in each study, and the diamond shape shows the severity of moral distress in nurses for the entire study

**Fig. 10:**
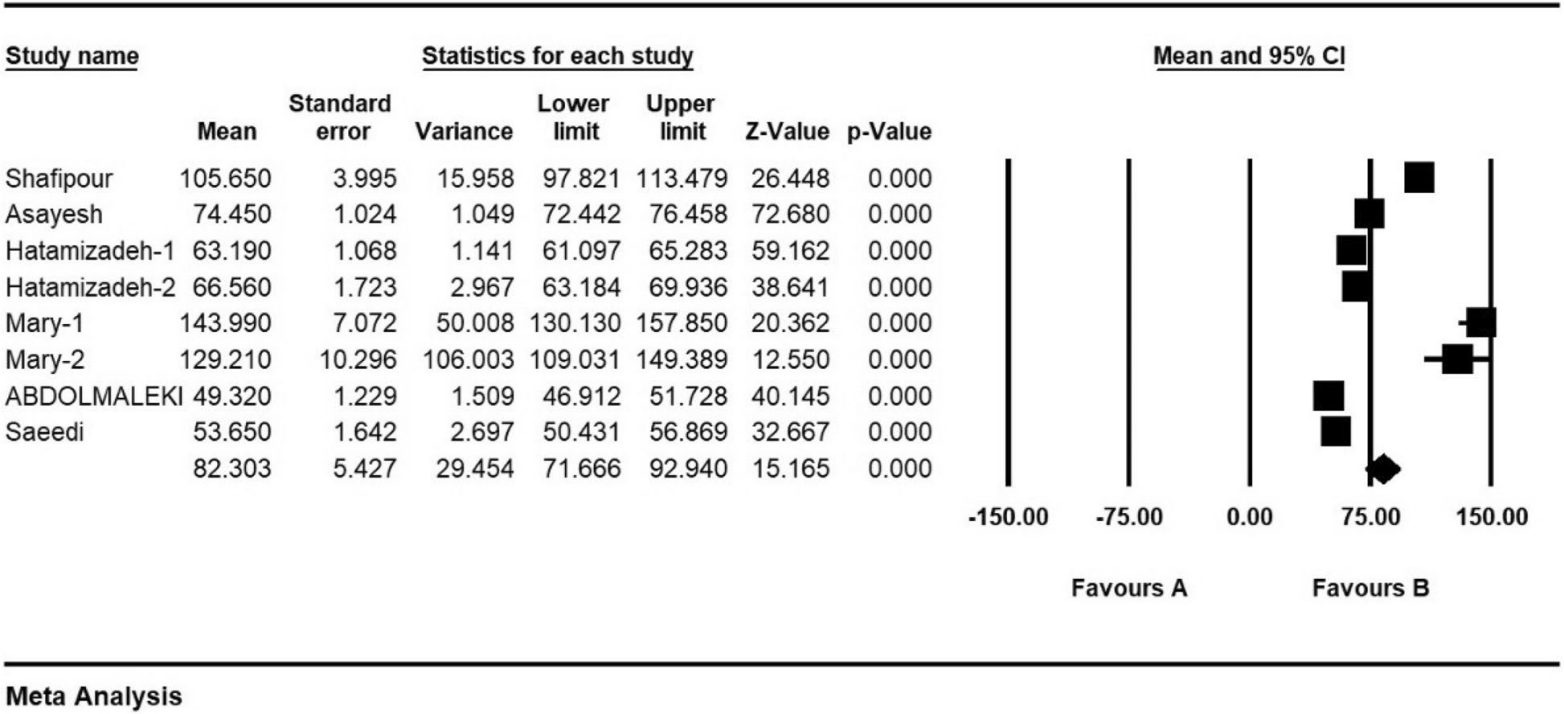
Severity of moral distress in nurses and 95% confidence interval in group (0–216). The middle point of each line segment shows the severity of moral distress in each study, and the diamond shape shows the severity of moral distress in nurses for the entire study

The severity of moral distress in 4 articles with a sample size of 1116 persons was 1.7 ± 0.37 from (0–4), in 5 articles with a sample size of 1282 persons, 2.6 ± 0.28 from (0–5), in 5 articles with a sample size of 944 persons, 3.9 ± 0.63 (0–6) and 8 articles with a sample size of 901 persons was 82.3 ± 5.4 (0–216) (Figs. 7, 8, 9 and 10).

## Discussion

The concept of moral distress has been introduced in the nursing literature for more than four decades and has been considered by researchers in this field. Historically, what caused the familiarity of nursing researchers with this concept is the work of Andrew Jameton, an American philosopher and researcher in the field of biological ethics, in 1984. This philosopher viewed moral distress from the ontological and epistemological perspectives. In the nursing literature, moral distress has been recognized as a major problem that nurses face in all care systems [76]. Therefore, providing care in clinical settings for nurses is associated with the phenomenon of moral distress [77]. This study aimed to determine the frequency and severity of moral distress in nurses in a systematic review and meta-analysis study.

The frequency score and severity of distress obtained from the whole scale were classified into four categories: (1–0), (1.01–2), (2.01–3), and (3.01–4) [17].

In the current systematic review and meta-analysis study, the frequency of moral distress was 1.7 ± 0.5, and the severity of moral distress was 1.7 ± 0.37 from (0–4). Also, in the present study, the frequency of moral distress was 3.07 ± 0.1, and the severity of moral distress was 2.6 ± 0.28 (0–5). The frequency score and severity of distress obtained from the whole scale were divided into five categories: (1–0), (1.01–2), (2.01–3), (3.01–4), and (4.01–5) [18] and also the frequency of moral distress was 3.2 ± 0.29 and severity of moral distress was 3.9 ± 0.63 (0–6). The score of frequency and severity of distress obtained from the whole scale are divided into three categories: low (0–2), medium (2.01–4), and high (4.01–6) [19]. Also, in the present study, the frequency of moral distress was 4.6 ± 0.518 from (1–7). The distress frequency score obtained from the whole scale is classified into three categories: (1–3), (3.01–5), and (5.01–7) [14]. The frequency of moral distress was 81.1 ± 4.6, and the severity of moral distress was 82.3 ± 5.4 (0–216) in a systematic review and meta-analysis study. The frequency score and severity of distress obtained from the whole scale are divided into three categories: (0–72), (144–73), and (145–216) [20]. According to the above scales, the frequency and severity of moral distress were moderate.

In a meta-analysis conducted by Yekta Kooshali (2018) in Iran, the severity of nurses’ moral distress was 2.23 (95% CI: 1.76–2.52) from 4, 2.95 (95% CI: 3–47. 2–43) from 5 and 3.43 (95% confidence interval: 3.54–3.32) from 6 and its frequency 1.92 (95% CI: 2–29-1.55) from 4, 3.02 (95% CI: 3.60–2.45) from 5 and 2.62 (95% CI: 2.73–2.52) [78]. This is consistent with the present study.

Pathologically, moral distress in nurses manifests despite transparency and stems from difficulty expressing concerns in such a way that they are heard [79, 80]. Additionally, neglected or marginalized nurses as a group of health care employees are likely to become disabled and avoid their ethical responsibilities [81].

It should also be said that when experiencing moral distress, nurses still know whether doing something is morally right. However, hospital organizational pressures, rules, regulations, and collegial conflict can still create obstacles. In this case, nurse performance will be impaired due to increased environmental stress [78–81].

Moral distress leads to a psychological imbalance in nurses when they believe in right and moral acts but are unable to carry them out [82]. In general, ethical distress for nurses has undesirable consequences [83]. Studies have shown that moral distress leads to job burnout [84, 85], reduces job satisfaction [23], and introduces challenges to professional independence [86]. Research in North America concluded that nurses who experience moral distress tend to leave service, whereas those who don’t are more likely to continue [73, 87]. The important point is that ethics-oriented nurses can experience less moral distress by linking personal ethical values and organizational ethics [88]. In this regard, the most important preventive factors of ethical distress are managerial support, professional independence, and mandated descriptions of specific tasks [89].

Moral distress is the emotional state that arises from a situation when a nurse feels that the ethically correct action to take is different from what they are tasked with doing. When policies or procedures prevent a nurse from doing what they think is right, that presents a moral dilemma [90–92].

When nurses experience moral distress, it’s important that they feel supported. They have to be able to address the issue in a safe and non-judgmental space [90–92].

Depending on the organization, nurses may be able to reach out to their ethics committee or even page an ethicist when a moral distress situation arises. These trained ethicists can help nurses find their voice and talk through their feelings and symptoms. The moral distress consult may help mitigate the nurse’s symptoms, assist the nurse in developing resilience, and strengthen their ethical confidence to deal with these feelings [90–92].

Several approaches to reducing moral distress have been published. Although further research is necessary to determine the effectiveness of these approaches, their foundations are solid, and they are, at least in part, useful to nurses at the bedside. The nurses can tailor the strategies described below to an appropriate individual, unit, or organizational setting [90–92].

The American Association of Critical Care Nurses has targeted moral distress as a priority area and has developed the 4 A’s approach to address and reduce moral distress. The 4 A’s are ASK (ask yourself whether what you are feeling is moral distress?), AFFIRM (What aspect of your moral integrity is being threatened?), ASSESS (What do you think is the “right” action and why is it so?), and ACT (Create a plan for action and implement it) [90–94].

According to the results of the present systematic review and meta-analysis, it is recommended that health system policymakers evaluate and plan continuously to improve nurses’ ethical levels to increase satisfaction and improve community health. Continuing classes of professional ethics appropriate to the culture of nations, appropriateness of educational content to the professional needs of nurses, considering shifts appropriate to nurses’ rest, and setting up group recreational programs can be the recommended strategies for policymakers.

Appropriate policy-making should also be considered in the discussion of hospital management, transparent hospital management, providing appropriate solutions to maintain ethics in the organization, as well as the possibility of consulting with the hospital management and the hospital council to make decisions on ethical issues, training classes, and solution skills. Hospital nurses and their management levels can also consider the issue of dealing with ethical problems properly.

## Limitations

One of the limitations of this study was that some samples were not randomly selected. Lack of uniform reporting, non-uniformity of the method, lack of consistency, and lack of full text of the papers presented at the conference can be mentioned.

## Conclusion

According to the present study and analysis based on various indicators, it was reported that moral distress among worshipers is a serious and significant problem. Due to the high level of moral distress in nurses, which affects their performance and will cause them to burn out, it is necessary for health policymakers and hospital managers to pay attention to this problem in nurses and provide training classes to reduce stress in nurses and to improve their performance, how to deal with ethical issues, as well as transparent management based on ethical rules in hospitals should be considered.

## Data Availability

Datasets are available through the corresponding author upon reasonable request.

## Abbreviations

SID: Scientific Information Database
WoS: Web of Science
STROBE: Strengthening the reporting of observational studies in epidemiology
PRISMA: Preferred Reporting Items for Systematic Reviews and Meta-Analysis
